# Dietary Supplements and Musculoskeletal Health and Function

**DOI:** 10.3390/nu15204404

**Published:** 2023-10-17

**Authors:** Gregory C. Bogdanis, Christoforos D. Giannaki

**Affiliations:** 1 School of Physical Education and Sport Science, National and Kapodistrian University of Athens, 17237 Athens, Greece; 2 Department of Life Sciences, University of Nicosia, 2417 Nicosia, Cyprus; giannaki.c@unic.ac.cy

This Special Issue of *Nutrients* ‘Dietary Supplements and Musculoskeletal Health and Function’ provides new insights into the use of a wide range of dietary supplements, such as zinc, creatine, Vitamin D, HMB, BCAA, betaine, glucoraphanin, citrulline and collagen, to improve bone and muscle structure and function. This Special Issue includes 17 papers in total, of which 13 are original articles and 4 are review articles.

The efficacy of Vitamin D supplementation as a treatment option for diffuse suffering syndromes, such as Fibromyalgia syndrome and chronic widespread musculoskeletal pain, was examined in a systematic review by Lombardo et al. [1]. The results revealed a therapeutic role of Vitamin D in terms of pain reduction in persons suffering from diffuse suffering syndromes. The effects of Vitamin D supplementation on bone mineral density (BMD) in kidney transplant recipients over a 2 year follow-up period were examined in an original study by Battaglia and colleagues. Long-term kidney transplant recipients exhibit Vitamin D insufficiency and reduced BMD. The findings did not show a positive effect of the supplementation on BMD parameters, as assessed using dual-energy X-ray absorptiometry (DEXA) [2].

Dietary supplements are very popular among athletes in terms of improving exercise and sports performance and enhancing health-related parameters [3]. Creatine is one of the most effective and well-studied supplements for athletic populations. Bogdanis and colleagues [4] showed an ergogenic effect of oral creatine supplementation during repeated treadmill sprinting, as shown by the lower drop in mean power and speed in the second half of the repeated sprint running protocol. Interestingly, this improvement was due to the higher power output and running speed in the last 5 s of each 10 s sprint. Another supplement that was studied for its ergogenic effect was L-citrulline supplementation. Theodorou and colleagues [5] investigated the effect of acute L-citrulline supplementation on nitric oxide bioavailability, inspiratory muscle oxygenation and respiratory performance in healthy males. Supplementation with 6 g L-citrulline increased nitric oxide bioavailability, but this did not have any effect on either respiratory performance or inspiratory muscle oxygenation. The potential ergogenic effect of dietary olive oil on mice endurance capacity and muscle metabolism was investigated by Komiya and colleagues [6]. The findings showed that dietary olive oil intake in mice significantly improved endurance capacity, which was mediated by increased intramuscular triacylglycerol accumulation in the gastrocnemius muscle. 

Polyphenols are known to have antioxidant and anti-inflammatory properties, and thus, can be used by athletes to enhance recovery after exercise training [7]. There is also evidence that polyphenol supplementation may improve sports and exercise performance [8]. Martinez-Canton et al. [9] investigated the effects of mango leaf extract (Zynamite PX^®^) combined with Quercetin supplementation on skeletal muscle NRF2 protein levels and NRF2-induced signaling under basal conditions and in response to high-intensity exercise in humans. The supplementation period lasted 48 h, and the supplementation group exhibited significant changes in resting skeletal muscle signaling, resembling those described after exercise training, and partly abrogated the stress kinase responses to exercise, as observed in trained muscles.

Curcumin is another polyphenol that received significant interest from the scientific community in recent decades due to its potential positive effects on human health. Deane and colleagues [10] investigated the effects of acute curcumin supplementation on cerebral and leg macrovascular blood flow, leg muscle microvascular blood flow, brachial artery endothelial function and leg insulin and glucose responses in healthy older adults. The findings of this study revealed that acute curcumin supplementation enhanced tibialis anterior microvascular blood volume without potentiating vastus lateralis microvascular blood volume, muscle glucose uptake or systemic endothelial or macrovascular function in healthy older adults, unveiling the promising effect of Curcumin on microvascular blood flow.

Recent studies suggest that betaine supplementation can play an ergogenic aid role as it has been reported to increase muscle strength and power in trained individuals [11]. Machek and colleagues examined whether a 28-day washout period was sufficient for serum betaine concentrations to return to baseline following a supplementation period of 14 days [12]. Serum betaine concentration was found to be significantly elevated immediately following the supplementation period and declined to statistically nonsignificant levels 4 days post-supplementation, suggesting that a more conservative 7 day washout period is sufficient to truly return both serum and skeletal muscle betaine content to pre-supplementation levels.

Zavros et al. evaluated the effect of eight weeks of Zinc and Selenium co-supplementation on resting metabolic rate (RMR), thyroid function, physical fitness and functional capacity in overweight and obese people under a hypocaloric diet [13]. The results showed that Zing and Selenium co-administration enhanced RMR (increased from 1923 ± 440 to 2364 ± 410 kcal/day) and increased Selenium serum levels and performance on the timed-up-and-go test without affecting thyroid hormones, body composition and physical fitness. 

Polyunsaturated fatty acids (PUFAs) have attracted considerable attention for their promising role in improving various health and quality of life parameters in many groups of the population, including older adults [14]. Felix-Soriano and coworkers [15] investigated the effect of docosahexaenoic acid (DHA)-rich n-3 fatty acid supplementation with or without resistance training in a randomized, double-blind, placebo-controlled trial in overweight/obese postmenopausal women. The DHA-rich supplement had beneficial effects on cardiovascular health markers in overweight/obese postmenopausal women, including a reduction in diastolic blood pressure and circulating triglycerides and increased muscle quality in the lower limbs. Notably, no synergistic effects were observed for DHA supplementation or resistance exercise combinations in the present study.

There is a growing interest in ketogenic diets and their effects on various physiological parameters in healthy and diseased populations. Yapukova and colleagues [16] shed light on the effects of ketogenic diets on skeletal muscle metabolism. The authors critically discussed some favorable adaptations of these diets to muscle metabolism-related parameters and especially energy metabolism, including fat and carbohydrate oxidation. However, they highlighted the fact that these diets could also induce adverse effects such as cardiac fibrosis, and thus must be followed with caution.

Recent epidemiological data show that sarcopenia, a condition characterized by a progressive loss of muscle mass and function, affects 10–16% of the elderly population worldwide [17]. In a review by Romani and colleagues [18], mitochondrial dysfunction, oxidative stress and inflammation were presented as key factors leading to sarcopenia, and the positive effects of branched amino acids, omega-3 PUFA and selected micronutrients on the above pathways were also discussed. Pereira et al. [19] examined the serum biomarker changes in response to 12 weeks of oral supplementation with Vitamin D and HMB in malnourished community-dwelling older adults with sarcopenia. Thirteen biomarkers significantly changed in response to the supplementation, which may be linked to improvements in skeletal muscle health. Interestingly, one of the articles of this Special Issue showed that a nutraceutical (Genistein–Lycopene combination) can reduce bone damage caused by chronic glucocorticoid therapy which induces osteoporosis [20]. These findings bear high clinical significance, as glucocorticoids are a first-line treatment option for immune-mediated and allergic diseases. However, glucocorticoids are frequently associated with adverse effects, including osteoporosis, and the current supplement may prevent these adverse effects.

Bone health may also be threatened by high cholesterol levels, which correlate with decreased bone density. Li and coworkers [21] examined Arctiin supplementation as an approach to prevent high cholesterol diet-induced bone loss by decreasing oxidative stress. Arctiin attenuated 7-KC-induced osteoclastogenesis by increasing the expression of reactive oxygen species scavenging genes in the Nrf2/HO-1/catalase signaling pathway, thereby decreasing osteoclast autophagy. The effects of glucoraphanin (GRA) on osteogenesis in human mesenchymal stromal cells were investigated by Gambari et al. [22]. The results showed that using cruciferous derivatives as natural alternatives to chemical hydrogen sulfide donors may improve osteogenesis and may be used for the treatment of bone-wasting diseases.

Osteoarthritis is a joint disease with a considerably high prevalence, affecting around 7% of the global population. Martínez-Puig et al. [23] examined the current collagen types that are prescribed as dietary supplements to improve joint health, elaborating on their mechanism of action and preclinical and clinical evidence. Both native and hydrolyzed collagen are effective and safe in terms of improving joint health. These findings are important as osteoarthritis is the main cause of years lived with disability worldwide, and it seems promising that patients with osteoarthritis may benefit from collagen supplementation. However, the authors note that further research is needed to obtain a clearer view of the use of each collagen type and composition for the various forms of osteoarthritis.

In summary, a wide variety of dietary supplements play a vital role in the improvement of musculoskeletal health and function in both diseased and healthy populations.
